# A Glaucoma-Associated Variant of Optineurin, M98K, Activates Tbk1 to Enhance Autophagosome Formation and Retinal Cell Death Dependent on Ser177 Phosphorylation of Optineurin

**DOI:** 10.1371/journal.pone.0138289

**Published:** 2015-09-16

**Authors:** Kapil Sirohi, Asha Kumari, Vegesna Radha, Ghanshyam Swarup

**Affiliations:** Centre for Cellular and Molecular Biology, Council of Scientific and Industrial Research, Hyderabad, 500 007, India; Casey Eye Institute, UNITED STATES

## Abstract

Certain missense mutations in optineurin/OPTN and amplification of *TBK1* are associated with normal tension glaucoma. A glaucoma-associated variant of OPTN, M98K, induces autophagic degradation of transferrin receptor (TFRC) and death in retinal cells. Here, we have explored the role of Tbk1 in M98K-OPTN-induced autophagy and cell death, and the effect of Tbk1 overexpression in retinal cells. Cell death induced by M98K-OPTN was dependent on Tbk1 as seen by the effect of Tbk1 knockdown and blocking of Tbk1 activity by a chemical inhibitor. Inhibition of Tbk1 also restores M98K-OPTN-induced transferrin receptor degradation. M98K-OPTN-induced autophagosome formation, autophagy and cell death were dependent on its phosphorylation at S177 by Tbk1. Knockdown of OPTN reduced starvation-induced autophagosome formation. M98K-OPTN expressing cells showed higher levels of Tbk1 activation and enhanced phosphorylation at Ser177 compared to WT-OPTN expressing cells. M98K-OPTN-induced activation of Tbk1 and its ability to be phosphorylated better by Tbk1 was dependent on ubiquitin binding. Phosphorylated M98K-OPTN localized specifically to autophagosomes and endogenous Tbk1 showed increased localization to autophagosomes in M98K-OPTN expressing cells. Overexpression of Tbk1 induced cell death and caspase-3 activation that were dependent on its catalytic activity. Tbk1-induced cell death possibly involves autophagy, as shown by the effect of *Atg5* knockdown, and requirement of autophagic function of OPTN. Our results show that phosphorylation of Ser177 plays a crucial role in M98K-OPTN-induced autophagosome formation, autophagy flux and retinal cell death. In addition, we provide evidence for cross talk between two glaucoma associated proteins and their inter-dependence to mediate autophagy-dependent cell death.

## Introduction

Glaucomas are a complex, heterogeneous and multi-factorial group of neurodegenerative eye diseases, characterized by a progressive degeneration of retinal tissues, particularly retinal ganglion cells. It is a major cause of irreversible blindness worldwide. Many genetic as well as environmental factors are involved in glaucoma pathogenesis [1–3]. Elevated intra-ocular pressure (IOP) is a major risk factor; however, in many cases IOP is in normal range. Glaucomatous condition associated with normal IOP is termed as normal tension glaucoma (NTG), a subset of primary open angle glaucoma (POAG). Six genes (*MYOC*, *OPTN*, *WDR36*, *NTF4*, *TBK1* and *ASB10*) have been linked with Mendelian forms of POAG [4–11]. Mutations in OPTN (*OPTN)* are associated with NTG and account for 1–2% of POAG [10]. Duplication of *TBK1* (encoding TANK binding kinase 1) has been reported in several populations [4, 6, 12]. Quite a few mutations in *OPTN* are reported in glaucoma, though only a few mutations, including E50K and M98K have been shown to alter cellular homeostasis and cause degeneration of retinal cells by engagement of distinct mechanisms [13–16]. The M98K mutation is more prevalent in Asian populations [17–19].

**Fig 1:**
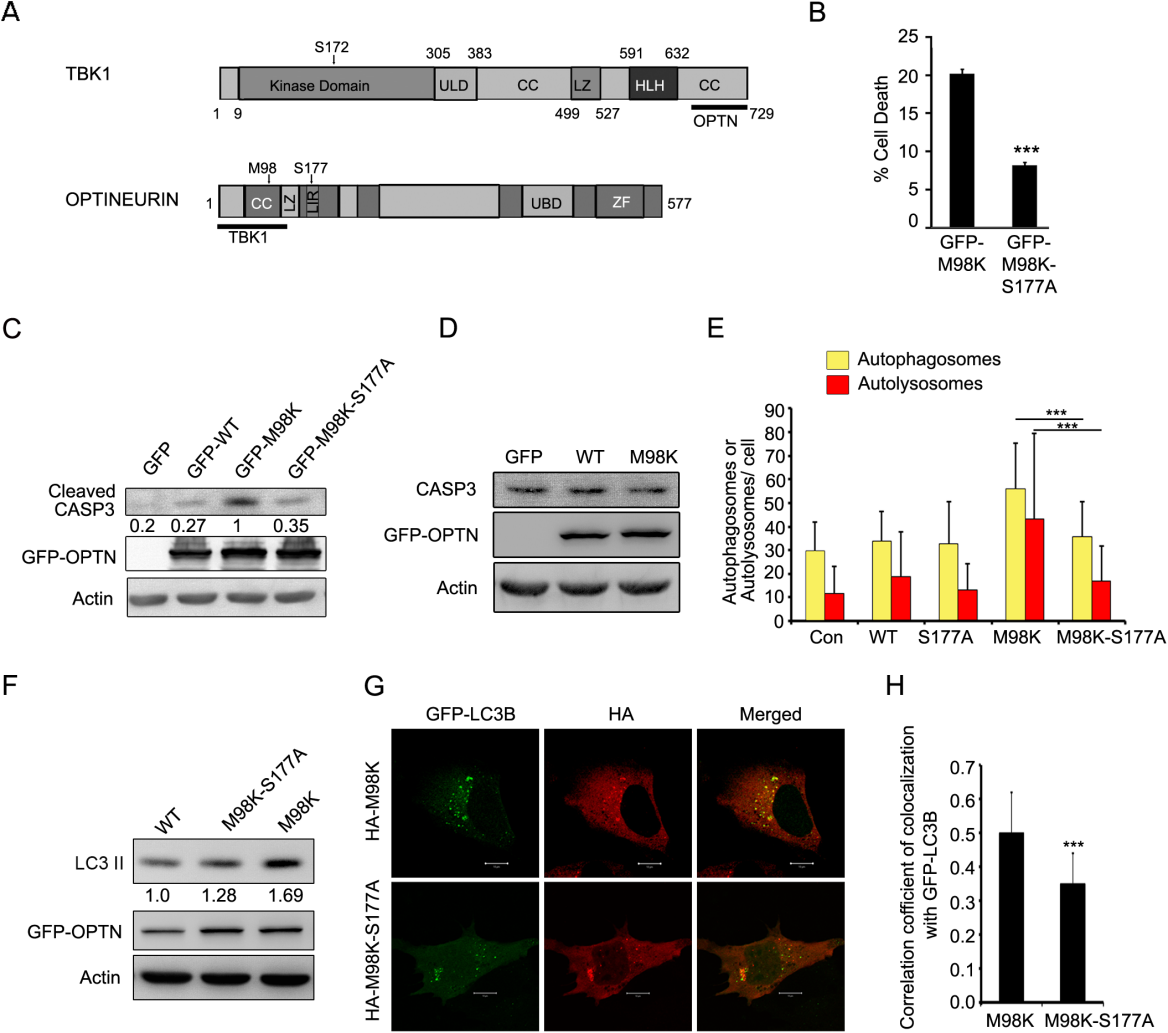
Phosphorylation of OPTN on Ser177 is important for M98K-OPTN-mediated autophagy and cell death. **(A)** Domain architecture of TBK1 and OPTN indicating their interacting regions. OPTN-binding site in TBK1 (689-729aa) and TBK1-binding site in OPTN (1-127aa) are shown by a thick bar. CC, coiled-coil; HLH, helix-loop-helix; LIR, LC3-interacting region; LZ, leucine zipper; UBD, ubiquitin-binding domain; ULD, ubiquitin-like domain; ZF, zinc-finger. **(B)** Quantitation of cell death induced by GFP-M98K-OPTN and its phosphorylation defective-mutant (M98K-S177A-OPTN) in RGC-5 cells upon 32 hrs of expression. Data represent mean ± s.d of percentage of expressing cells showing apoptotic morphology after subtraction of cell death seen in non-expressing cells. n = 6, ***p < 0.001. **(C)** M98K-S177A-OPTN mutant shows reduced cleavage of caspase 3. Western blots showing cleavage of caspase 3 upon overexpression of GFP, GFP-WT-OPTN, GFP-M98K-OPTN and GFP-M98K-S177A-OPTN mutant in RGC-5 cells. Actin was used as a loading control. The numbers below cleaved caspase 3 blot indicate relative caspase 3 levels after normalization with actin. **(D)** M98K-OPTN mutant did not change expression of caspase 3. Western blots showing caspase 3 upon overexpression of GFP, GFP-WT-OPTN and GFP-M98K-OPTN mutant in RGC-5 cells. Actin was used as a loading control. **(E)** Phosphorylation of M98K-OPTN at Ser177 is required for autophagy induction. Quantitation of number of autophagosomes (yellow dots) and autolysosomes (red dots) per cell in control (con), HA-OPTN (WT), HA-S177A-OPTN, HA-M98K-OPTN or HA-M98K-S177A-OPTN transfected cells. n = 35 cells, ***p< 0.001. **(F)** M98K-S177A-OPTN mutant shows reduced LC3II levels. Western blots showing LC3II levels upon overexpression of GFP-WT-OPTN, GFP-M98K-S177A-OPTN and GFP-M98K-OPTN mutants in RGC-5 cells. Actin was used as a loading control. The numbers below LC3II blot indicate relative LC3II levels after normalization with actin. **(G)** Representative confocal images of RGC-5 cells expressing GFP-LC3B along with HA-M98K or HA-M98K-S177A. **(H)** The graph shows Pearson’s correlation coefficient of colocalization of LC-3B with M98K or M98K-S177A. n = 25 cells, ***p< 0.001.

OPTN is a 577 amino acid protein which is organized into distinct domains such as LC-3 interacting region (LIR), zinc finger (ZF), ubiquitin binding domain (UBD) and leucine zipper (LZ) (Fig 1A). OPTN is a multi-functional protein, and it generally acts as an adaptor by interacting with a variety of cellular proteins [20–22]. It is involved in several cellular processes such as vesicular trafficking, autophagy, mitosis, immune response and signal transduction [15, 22–31]. E50K mutation of OPTN is the most severe disease causing mutation. Transient expression of E50K induces death of RGC-5 cells, a retinal cell line but not of other cell lines tested [14]. Deleterious effects of E50K-OPTN mutant on retinal ganglion cells as well as on other retinal cells was also seen in E50K transgenic mice [32] suggesting the usefulness of this cell culture model [33]. E50K-OPTN-induced death of retinal cells is due to block in autophagy and defective transferrin receptor (TFRC) recycling; a GTPase activating protein, TBC1D17, plays an important role in these processes [13, 24, 34]. On the other hand, overexpression of M98K-OPTN alters TFRC recycling by inducing its autophagic degradation through the recruitment of RAB12 to autophagosomes [15]. In fact, M98K-OPTN overexpression induced autophagic cell death in retinal cells [15]. Reduced levels of TFRC upon M98K-OPTN overexpression seems to be the cause for RGC-5 death, as restoration of TFRC levels resulted in inhibition of M98K-OPTN-induced cell death [15]. However, we lack an understanding of how M98K-OPTN triggers retinal cells to undergo autophagy and degrade TFRC, which could ultimately lead to glaucoma pathology.

OPTN plays an important role in autophagy; it functions as an autophagy receptor, and is involved in degradation of ubiquitinated protein aggregates, damaged mitochondria and ubiquitinated bacteria [27, 35, 36]. It interacts with TBK1 (TANK binding kinase 1), another protein involved in autophagy [37]. TBK1 phosphorylates OPTN at Ser177 and this phosphorylation enhances its ability to bind to LC3, a protein essential for autophagosome formation, and sequesters autophagic cargo to autophagosomes [27]. E50K mutant of OPTN shows stronger interaction with TBK1 compared to wild-type OPTN [37, 38]. This stronger interaction results in E50K protein insolubility, which has been suggested to cause degeneration of RGCs associated with POAG [38].

TBK1 is a ubiquitously expressed protein composed of 729 amino acids with four functionally distinct domains—N-terminal kinase domain, a ubiquitin-like domain (ULD) and two putative C-terminal coiled coil regions, including a LZ and a helix-loop-helix motif (HLH) [39] (Fig 1A). The C-terminal coiled coil domain has also been named as the adaptor binding domain (AB) [40]. The ULD is required for functions such as kinase activation and substrate presentation [41]. TBK1 functions as a nodal protein in cellular signalling where it receives multiple upstream signals and modulates the function of numerous downstream targets. It mediates innate immunity signalling through NF-κB, IRF3 and IRF7 activation [42, 43] and regulates immune responses to bacterial and viral infections [44, 45]. TBK1 was shown to be involved in autophagy, having a role in clearance of ubiquitinated invasive bacteria where it phosphorylates autophagy receptors, and mediates pathogen clearance and inhibits its proliferation [27, 46]. TBK1 knockdown did not affect formation of autophagosomes but suppressed their maturation into autolysosomes [47].

Here, we explored the possibility of functional association of these two proteins with respect to glaucoma pathogenesis. We report that phosphorylation of M98K-OPTN at Ser177 by Tbk1 is required for M98K-OPTN induced cell death, autophagosome formation, autophagy flux and TFRC degradation in retinal cells. Moreover, compared to WT-OPTN, M98K-OPTN showed higher levels of Tbk1 mediated phosphorylation at Ser177 in retinal cells. We also show that overexpression of Tbk1 induces death in retinal cells, and that OPTN is required for Tbk1-induced cell death.

## Materials and Methods

### Cell Culture and Transfections

RGC-5 (provided by Dr. Neeraj Agarwal, University of North Texas Health Science Centre, Fort Worth, TX) [48], IMR-32 and HeLa (American Type Culture Collection) cells were cultured in Dulbecco’s Modified Eagle’s Medium (DMEM) containing 10% FCS and antibiotics in a humidified atmosphere of 5% CO_2_ at 37°C. Transfections were performed on cells grown on coverslips or cell culture dishes using Lipofectamine 2000 (Invitrogen, 11668029) or Lipofectamine 3000 (Invitrogen, L3000015) according to the manufacturer’s instructions. For amino-acid starvation, cells were washed thrice with PBS and kept in Earle’s Balanced Salt Solution (EBSS) medium (Invitrogen, 14155063) at 37°C for the required time.

### Expression Vectors

Plasmid vectors for expressing human OPTN and its mutants (M98K and M98K-D474N) with HA and GFP-tags have been described [14, 15]. HA-S177A-OPTN and HA-M98K-S177A-OPTN mutants were made by site-directed mutagenesis from HA-OPTN and HA-M98K-OPTN respectively, following the protocol described in QuikChange Site-Directed Mutagenesis Kit (Stratagene/Agilent). S177A-OPTN and M98K-S177A-OPTN were cloned in pEGFP-C3 (Clontech, 6082–1) to produce GFP-tagged OPTN mutants. cDNA of mouse *Tbk1* was amplified by RT-PCR using RNA from RGC-5 cells and cloned in pcDNA3.1 and EGFP vectors. GFP-K38A-Tbk1 and HA-K38A-Tbk1 were made by site-directed mutagenesis from GFP-Tbk1 and HA-Tbk1 respectively. The plasmids, pDest-GFP-LC3B and mCherry-GFP-LC3B were kindly provided by Dr. Terje Johansen (University of Tromsø, Tromsø, Norway) [49]. Adenoviral vectors expressing wild-type OPTN and its M98K, M98K-D474N and E50K mutants have been described earlier [15, 50].

### Construction of Vectors Expressing shRNAs

Plasmid vectors for expressing shRNAs directed against mouse *Tbk1* and *Optn* were constructed using a U6 promoter-based vector (pmU6) as described previously [51–53]. The mouse *Tbk1* sequences targeted by ShA, ShB and ShC were from nucleotides 1780 to 1798, 2787 to 2805, and the 1644 to 1662 (GenBankTM accession NM_019786.4). The mouse *Optn* sequences targeted by ShA and ShB were from 287 to 305, and 1109 to 1127 (GenBankTM accession NM_181848.4). A vector expressing shRNA of unrelated sequence (Con-Sh) of the same length was used as a control. The plasmid vectors for expressing shRNAs directed against mouse *Atg5* have been described [15].

### Antibodies and Reagents

Mouse monoclonal anti-TFRC antibody (Zymed, 136800), rabbit polyclonal cleaved CASP3 (Asp-175) antibody (Cell Signaling Technology, 9664), rabbit polyclonal anti-ATG5 antibody (Cell Signaling Technology, 8540), rabbit monoclonal anti-phospho-TBK/NAK (Ser172) antibody (Cell Signaling Technology, 5483), rabbit monoclonal anti-TBK1/NAK antibody (Cell Signaling Technology, 3504 and Abcam, ab40676), mouse monoclonal anti-HA antibody (Roche Applied Biosystems, 11583816001), rabbit polyclonal anti-HA (Santa Cruz, sc-805), rabbit polyclonal and mouse monoclonal anti-GFP (Santa Cruz, sc-9996, sc-8334), anti-GAPDH (Millipore, MAB374) and anti-actin (Millipore, MAB1501) are commercially available. Secondary antibodies used were Cy-3-conjugated anti-mouse IgG (Amersham, PA43002), Cy-3-conjugated anti-rabbit IgG (Amersham, PA43004), HRP conjugated anti-mouse IgG (Amersham, NA9310), HRP conjugated anti-rabbit IgG (Amersham, NA934), Alexa-488 anti-mouse and anit-rabbit IgG (Molecular Probes, A21202, A21206). Phospho-OPTN antibody specific to S177 residue of OPTN was a generous gift from Dr. Ivan Dikic of Goethe University Medical School, Theodor-Stern-Kai 7 60590 Frankfurt am Main / Germany [27]. BX-795 (Calbiochem, 204001) and chloroquine (Sigma, C6628) are commercially available.

### Indirect Immunofluorescence and Confocal Microscopy

For immunofloresence analysis, cells were grown on coverslips and were transfected with the required plasmids. At the indicated time, cells were fixed with 3.7% formaldehyde and then stained with appropriate antibodies. Indirect immunostaining of cells and microscopy was carried out essentially as described previously [24, 54]. All images were captured with LSM 510 Meta NLO Confocal Microscope from Carl Zeiss (Jena, Germany) using 63X oil immersion objective lens (NA 1.4). For colocalization, two, 0.33 μm, optical Z-sections through the centre of the cells were projected and was analyzed by using LSM 510 (version 3.2) software. Pearson’s correlation coefficients for colocalization were calculated by LSM 510 software. Nikon Eclipse Ni microscope (Japan) was also used for observing immunofluorescence. Images were captured using the Axiocam (Zeiss) CCD camera and processed with Axiovision 4 software. All images were assembled using Adobe Photoshop.

### Measurement of Autophagic Flux

For measuring the number of autophagosomes and autolysosomes, cells grown on coverslips were transfected with mCherry-GFP-LC3B reporter construct along with required plasmids. The fixed cells were imaged using a confocal microscope and the number of autophagosomes (green dots and red dots, which show yellow colour in merged image) and autolysosomes (red dots) was quantified as described previously [15, 49]. The protein expressed by mCherry-GFP-LC3B construct goes to autophagosomes as well as autolysosomes. Autophagosomes appear as yellow dots due to merging of red and green fluorescence, whereas, the autolysosomes appear as red dots because of quenching of GFP signal due to low pH.

### Co-Immunoprecipitation and Western Blotting

RGC-5 cells were seeded in 35-mm dishes and after 20 hrs cells were infected with required adenoviruses. After 24 hrs, cells were washed with ice-cold PBS followed by lysis at 4°C for 20 mins in lysis buffer (25 mM Tris-HCl, pH 7.4, 150 mM NaCl, 0.8% Triton X-100, 1 mM PMSF, 0.1% BSA, 5 mM EDTA and protease inhibitor cocktail). Lysates were centrifuged at 10,000g for 15 mins at 4°C. Supernatant fraction was used for immunoprecipitation using the required antibody or normal IgG as a control antibody (2 μg of either antibody). Supernatants were incubated at 4°C for 8–10 hrs with antibodies and with protein A/G plus agarose beads (20 μl) for another 2 hrs. The beads containing immune complexes were given three washes with 400 μl wash buffer (20 mM HEPES pH 7.4, 0.1% Triton X-100, 150 mM NaCl, 10% glycerol, 1 mM PMSF, and protease inhibitors) in each wash, and then boiled in sample buffer for SDS-PAGE [55]. The samples were resolved on SDS-PAGE and transferred to a nitrocellulose membrane for western blot analysis as described previously [54].

### Cell Death Assays

Cells expressing the required proteins were stained with antibodies to detect expression or visualized using GFP tag. Cells were mounted in Dapi containing mountant to visualize nuclear morphology. Cells showing changes in morphological features like loss of refractility, cytoplasmic shrinkage and condensed chromatin were scored as dead cells [14, 15]. Quantitative analysis of apoptotic cells was carried out as described previously [14, 15]. Cells not expressing the transfected protein were also scored for cell death from each coverslip and cell death in these non-expressing cells were 2–3%. The cell death data shown in various figure represents cell death in expressing cells after subtracting cell death in non-expressing cells.

### Statistical Analysis

Bar diagrams represent mean ± s.d values. Differences between means were tested using Student’s t-test.

## Results

### Phosphorylation at Ser177 Is Required for M98K-OPTN-Induced Autophagosome Formation, Autophagy Induction and Cell Death

To explore the molecular mechanisms of glaucoma pathogenesis, RGC-5 cell line has been used in several studies [14, 16, 25, 33, 56, 57]. This cell line, which was originally described as a rat retinal ganglion cell line, has been re characterized [33, 58–60] and it shows markers of neural precursor cells, but presence of markers of retinal ganglion cells has been controversial. We have characterized RGC-5 cell line by carrying out RT-PCR analysis for expression of relevant genes. Our results show that this cell line expresses markers of neuronal cells (Map-1b and βIII-tubulin) and neural precursor cells (nestin) (S1 Fig). Expression of nestin in RGC-5 cells was high compared to mouse retina and it decreased upon differentiation with forskolin (S1 Fig). We observed very low level expression of Thy1, a marker for retinal ganglion cells, which increased slightly upon differentiation by forskolin (S1 Fig). We have also cloned and sequenced cDNAs coding for optineurin and TBC1D17 using RNA from RGC-5 cells, which has confirmed that the cell line we use is definitely of mouse origin. Therefore, we conclude that the RGC-5 cell line we have used is a neural precursor cell line derived from the mouse retina. Our results are in agreement with others [58, 59], who reached the same conclusion. RGC-5 is probably the same as 661W [58, 60] photoreceptor cell line. Morphologically, these cells show neuronal phenotype with long protrusions (neurites) upon differentiation (S1 Fig).

We have shown earlier that overexpression of M98K-OPTN induces autophagic degradation of TFRC and cell death selectively in RGC-5 cells [15]. This cell death is dependent on autophagy [15]. It is also known that phosphorylation of OPTN at Ser177 (S177) enhances its function as an autophagy receptor by increasing its interaction with the autophagosomal protein LC3 [27]. We examined if M98K-OPTN-induced cell death depends upon its phosphorylation at Ser177. Ser177 of M98K-OPTN was mutated to alanine by site-directed mutagenesis to make a phosphorylation-defective mutant, M98K-S177A. RGC-5 cells grown on coverslips were transiently transfected with GFP tagged M98K-OPTN or M98K-S177A-OPTN and after 32 hrs, scored for cell death in expressing and non-expressing cells. Transient expression of M98K-S177A-OPTN resulted in significantly less cell death compared to M98K-OPTN (Fig 1B). Compared to M98K-OPTN, M98K-S177A-OPTN overexpression showed reduced levels of cleaved caspase-3 (Fig 1C), an indicator of caspase-3 activation, suggesting that S177A mutant of M98K-OPTN was compromised in its ability to induce cell death. These constructs were expressed at similar levels (Fig 1C). There was no change in caspase-3 protein level upon overexpression of M98K-OPTN (Fig 1D).

In order to test the effect of M98K-OPTN phosphorylation at Ser177 on autophagy induction, we overexpressed M98K-OPTN and the phospho-defective mutant, M98K-S177A-OPTN, along with a reporter construct, mCherry-GFP-LC3B, and measured number of autophagosomes and autolysosomes. Since GFP is sensitive to low pH of lysosomal lumen, autolysosomes appear as red dots while autophagosomes appear as yellow dots due to the colocalization of GFP and mCherry. M98K-S177A-OPTN expressing cells showed less number of autophagosomes and autolysosomes compared to M98K-OPTN expressing cells, suggesting that M98K-S177A mutant was defective in inducing autophagosome formation and autophagy flux (Fig 1E and S2 Fig). We observed that M98K-OPTN expressing cells showed increased levels of LC3II protein compared to WT-OPTN-expressing cells and M98K-S177A-OPTN-expressing cells showed reduced level of LC3II protein compared to M98K-OPTN-expressing cells (Fig 1F). To test the effect of phosphorylation of M98K-OPTN at Ser177 on its ability to colocalize with LC3, we coexpressed M98K-OPTN or its phospho-defective mutant, M98K-S177A-OPTN with GFP-LC3B. M98K-S177A-OPTN showed less colocalization with GFP-LC3B, an autophagosome marker, compared to M98K-OPTN in vesicular structures likely to be autophagosomes (Fig 1G and 1H). These results indicate that phosphorylation of M98K-OPTN at Ser177 is critical for its ability to induce autophagosome formation and autophagy activity.

### Role of OPTN in Autophagosome Formation

**Fig 2:**
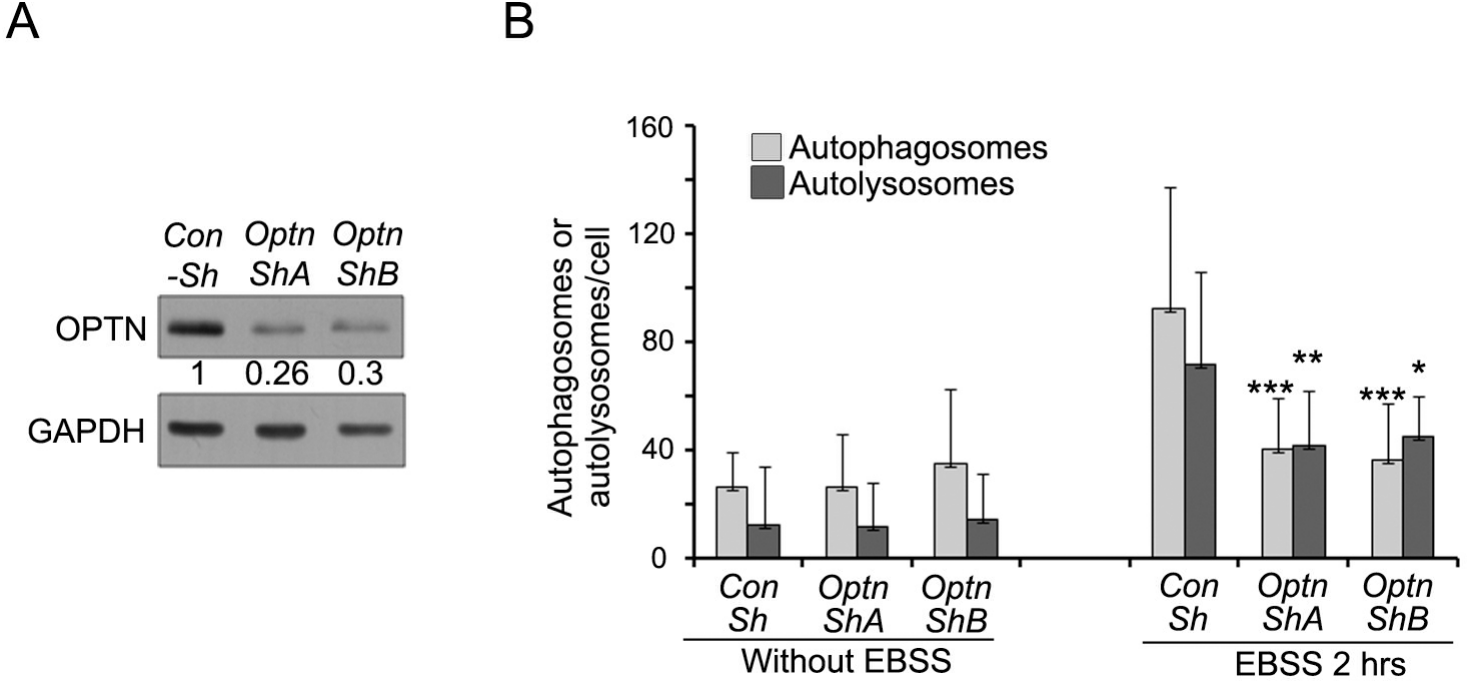
Knockdown of OPTN decreases starvation induced formation and maturation of autophagosomes. **(A)** Western blot shows knockdown of endogenous OPTN by shRNAs, shA and shB in RGC-5 cells. GAPDH was used as a loading control. Con-sh, control shRNA. **(B)** Quantitation of autophagosomes (yellow dots) and autolysosomes (red dots) per cell upon expression of m-cherry-GFP-LC3 along with control ShRNA (Con-sh) or OPTN shRNA, shA and shB in untreated conditions and in amino acid and serum-starved condition (EBSS for 2 hrs). n = 50 cells, ***p < 0.001.

M98K-OPTN expression induced autophagy, which could be due to gain of a new function by M98K mutation or due to enhancement of normal function of OPTN in autophagosome formation and maturation. This prompted us to investigate the role of OPTN in autophagosome biogenesis and autophagosome maturation. Knockdown of *OPTN* using two plasmid constructs expressing shRNA, ShA and ShB, resulted in reduced autophagosome and autolysosome numbers upon induction of autophagy by amino acid starvation (Fig 2A and 2B). However, under basal conditions, knockdown of OPTN did not reduce the number of autophagosomes or autolysosomes (Fig 2A and 2B). The level of OPTN in knockdown cells may be sufficient to support autophagosome formation under basal condition when autophagy activity is low, but may not be sufficient when autophagy is increased by starvation.

### M98K-Mutant Shows Enhanced Tbk1 Dependent Phosphorylation at Ser177

**Fig 3:**
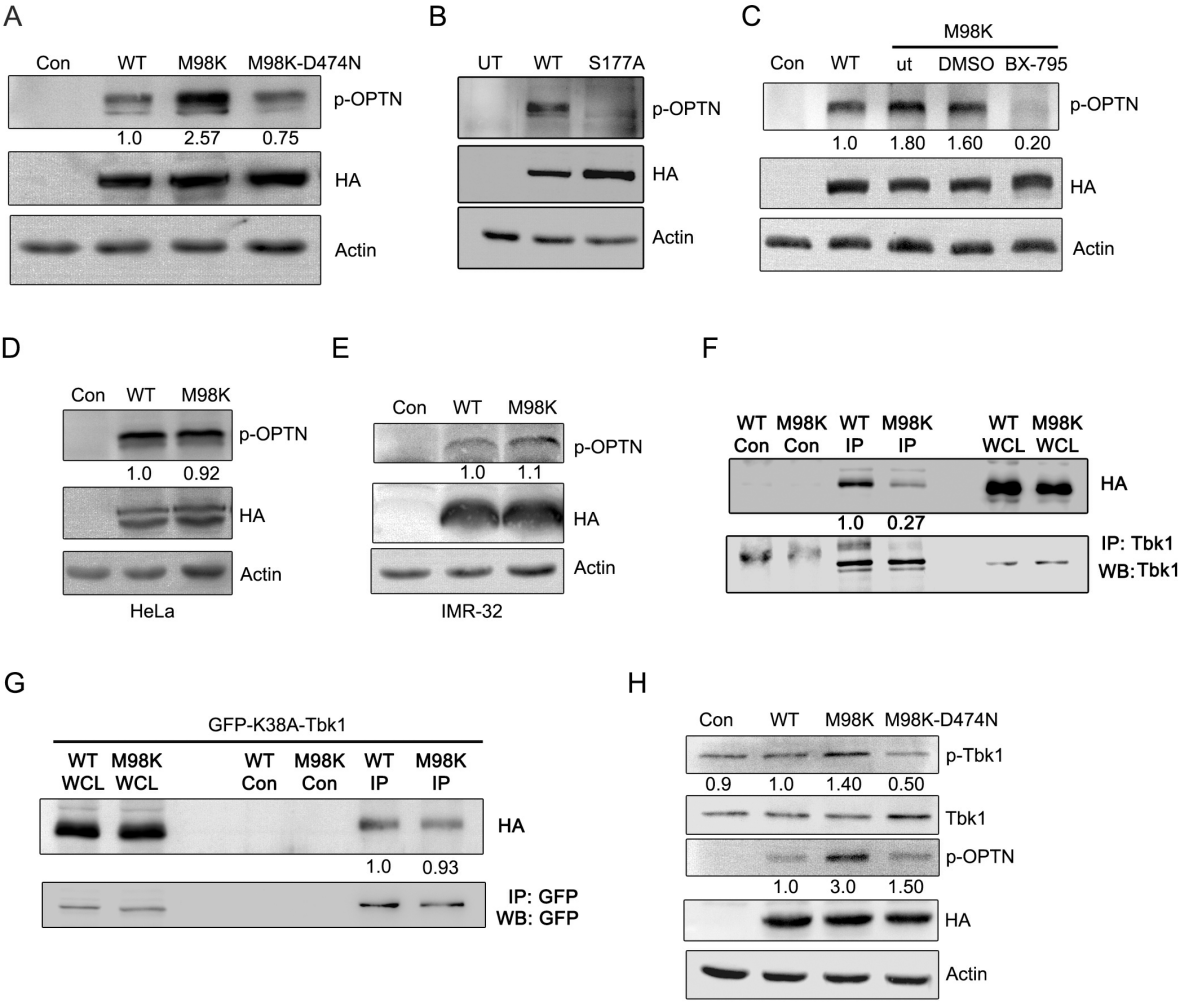
M98K-OPTN shows enhanced Tbk1 dependent phosphorylation. **(A)** Western blots show p-OPTN (p-S177) levels in RGC-5 cells infected with control (con), WT-OPTN, M98K-OPTN or M98K-D474N-OPTN adenoviruses after 24 hrs of expression. HA blot shows expression levels of various constructs of OPTN. Actin was used as a loading control. The numbers below p-OPTN blot indicate relative p-OPTN levels after normalization with OPTN expression blot. **(B)** Western blots show p-OPTN (Ser177) levels in RGC-5 cells transfected with WT-OPTN or S177A-OPTN expression vector after 24 hrs of expression. UT, untransfected. **(C)** Inhibition of Tbk1 reduces M98K-OPTN phosphorylation. RGC-5 cells were infected with control (con), WT-OPTN or M98K-OPTN adenoviruses; M98K adenovirus infected cells were either kept untreated (ut) or treated with DMSO or with 1 μM BX-795 for 18 hrs. Cell lysates were then subjected to western blotting. **(D and E)** Expression of M98K-OPTN in HeLa or IMR-32 cells does not result in enhanced phosphorylation. Relative p-OPTN levels in Hela and IMR-32 cells respectively, after control (con) WT-OPTN or M98K-OPTN adenovirus infection. **(F)** Interaction of OPTN with Tbk1 in retinal cells. RGC-5 cells were infected with adenoviruses expressing HA-tagged WT-OPTN or M98K-OPTN for 24 hrs and cell lysates were subjected to immunoprecipitation with Tbk1 antibody or control antibody (Con) and western blotting done with HA and Tbk1 antibodies. WCL, whole cell lysate. The numbers below HA blot indicate relative levels of co-purified WT-OPTN and M98K-OPTN levels after normalization with immunoprecipitated Tbk1 protein. **(G)** Interaction of catalytically inactive mutant of Tbk1, K38A-Tbk1 with WT-OPTN and M98K-OPTN in retinal cells. RGC-5 cells were co-transfected with plasmids expressing HA-tagged WT-OPTN or M98K-OPTN along with GFP-tagged K38A-Tbk1 for 24 hrs and cell lysates were subjected to immunoprecipitation with GFP antibody or control antibody (Con) and western blotting done with HA and GFP antibodies. WCL, whole cell lysate. The numbers below HA blot indicate relative levels of co-purified WT-OPTN and M98K-OPTN levels after normalization with immunoprecipitated K38A-Tbk1 protein. **(H)** M98K-OPTN activates Tbk1. Western blots show p-Tbk1 (Ser172) levels in RGC-5 cells infected with control (con), WT-OPTN, M98K-OPTN or M98K-D474N-OPTN adenoviruses after 24 hrs of expression. HA blot shows expression levels of various constructs of OPTN. Actin was used as a loading control. The numbers below p-Tbk1 blot indicate relative p-Tbk1 levels after normalization with Tbk1 blot. The number below p-OPTN blot indicates relative p-OPTN levels after normalization with expression blot.

Since phosphorylation of M98K at Ser177 is a prerequisite for cell death induced by M98K-OPTN, and phosphorylation on Ser177 potentiates autophagy ability, we further explored whether M98K-OPTN gets phosphorylated better than WT-OPTN. We analysed WT-OPTN and M98K-OPTN expressing cell lysates by western blotting using p-S177-OPTN specific antibody (antibody specific to phosphorylation at Ser177 of OPTN). M98K-OPTN expressing cells showed enhanced signal of p-S177-OPTN compared to WT-OPTN (Fig 3A). Interestingly, ubiquitin binding defective mutant of M98K-OPTN, M98K-D474N-OPTN, showed reduced phosphorylation suggesting requirement of UBD for differential phosphorylation. The S177A mutant of OPTN did not show any phosphorylation, showing the specificity of the antibody (Fig 3B). BX-795, a chemical inhibitor, is known to specifically inhibit TBK1 kinase activity [27, 61]. Treatment of M98K-OPTN expressing cells with, BX-795, inhibited M98K-OPTN phosphorylation at Ser177 indicating that phosphorylation of OPTN at Ser177 is mediated by Tbk1 (Fig 3C). Earlier, we have shown that M98K-OPTN induces cell death in retinal cells (RGC-5) selectively. In order to check the selectivity of this enhanced phosphorylation in other cell types, we overexpressed WT-OPTN and M98K-OPTN in HeLa and IMR-32 cells and found that there was no or very little difference in the phosphorylation of WT-OPTN and M98K-OPTN at Ser177 in these cell lines (Fig 3D and 3E), indicating the cell type selectivity in enhanced phosphorylation at Ser177 of M98K-OPTN. This was not due to lower level of TBK1 protein in HeLa or IMR-32 cells (S3 Fig).

### M98K-OPTN Activates Tbk1

OPTN and TBK1 are present in a signalling complex and directly interact with each other in the cellular milieu to facilitate signalling [29, 37]. Since WT-OPTN and M98K-OPTN differed in their ability to be phosphorylated by Tbk1, we examined if this was due to difference in interaction between WT-OPTN and M98K-OPTN with Tbk1. RGC-5 cells were infected with adenoviruses expressing HA-tagged WT or M98K-OPTN for 24 hrs and cell lysates subjected to immunoprecipitation using Tbk1 antibody. Fig 3F shows that WT-OPTN and M98K-OPTN can be co-precipitated in a complex with cellular Tbk1. Interestingly, it was observed that lower levels of the M98K-OPTN co-precipitated with Tbk1, suggesting that M98K-OPTN shows weaker interaction with Tbk1 in these cells. The weaker interaction between Tbk1 and M98K-OPTN could be because M98K-OPTN readily gets phosphorylated compared to WT-OPTN and then the substrate and enzyme fall apart from each other. We also tested the interaction of inactive Tbk1 mutant, GFP-K38A-Tbk1 with HA-WT or HA-M98K-OPTN by immunoprecipitation (Fig 3G). Unlike the active enzyme the catalytically inactive Tbk1 does not differ in interaction with WT-OPTN or M98K-OPTN.

Since enhanced phosphorylation of M98K was not due to its ability to interact better with Tbk1, we investigated if M98K-OPTN expression activates Tbk1. Since Tbk1 gets activated when phosphorylated at Ser172 position in the kinase activation loop, we examined the levels of Ser172 phosphorylation on Tbk1 in M98K-OPTN expressing cells by using a phospho-Ser172 specific antibody. We overexpressed WT-OPTN, M98K-OPTN and M98K-D474N mutants in RGC-5 cells. M98K-OPTN expressing cells showed enhanced Tbk1 phosphorylation compared to cells expressing WT-OPTN or ubiquitin binding defective mutant of M98K, M98K-D474N (Fig 3H). These results suggest that expression of M98K-OPTN activates Tbk1 and function of UBD is required for Tbk1 activation by M98K-OPTN.

### Inhibition of Tbk1 Function Compromises M98K-OPTN-Induced Cell Death

**Fig 4:**
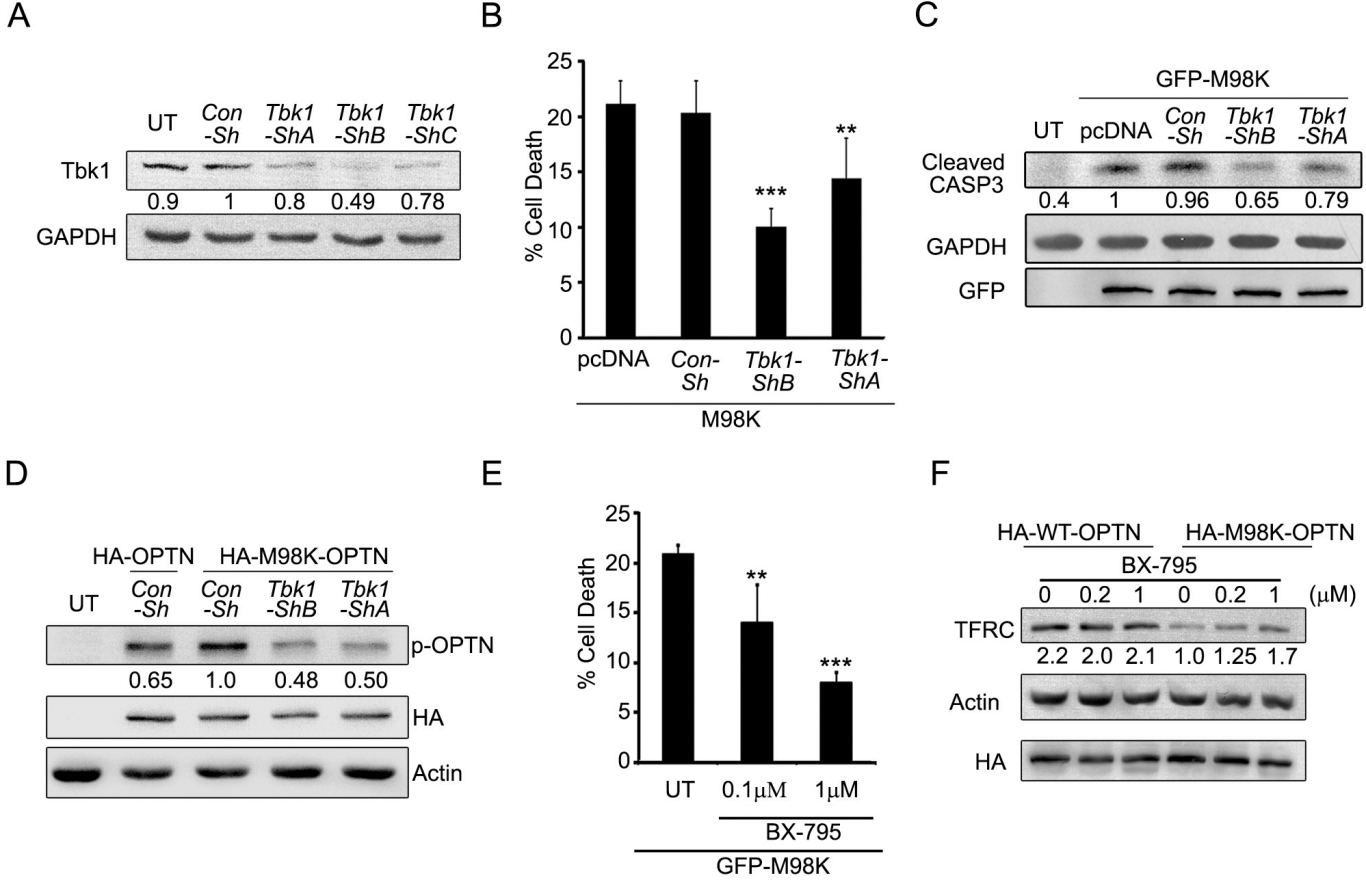
Tbk1 is required for M98K-OPTN-induced cell death and TFRC degradation in RGC-5 cells. **(A)** Knockdown of Tbk1 in RGC-5 cells. RGC-5 cells were transfected with either Con-sh, *Tbk1*-shA, *Tbk1*-shB or *Tbk1*-shC and after 30 hrs of expression cell lysates were made and subjected to western blotting. GAPDH was used as a loading control. The numbers below Tbk1 blot indicate relative Tbk1 levels after normalization. **(B)** Knockdown of Tbk1 reduced M98K-induced cell death. Data represent quantitation of cell death induced by M98K-OPTN with or without Tbk1 knockdown. n = 6, ***p < 0.001, **p<0.01. **(C)** Knockdown of Tbk1 reduces M98K-induced caspase 3 cleavage. Western blot shows cleavage of caspase 3 with or without Tbk1 knockdown. GAPDH was used as a loading control. The numbers below cleaved caspase 3 blot indicate relative cleaved caspase 3 levels after normalization. **(D)** Phosphorylation of M98K-OPTN is dependent on Tbk1. RGC-5 cells were transfected with either HA-OPTN or HA-M98K-OPTN along with indicated shRNAs, and after 30 hrs of expression, cell lysates were made and subjected to western blotting. Actin was used as a loading control. The numbers below p-OPTN blot indicate relative p-OPTN levels after normalization with expression blot, HA-OPTN. UT, untransfected. Con-Sh, control ShRNA. **(E)** M98K-OPTN-induced cell death of RGC-5 cells is dependent on kinase activity of Tbk1. RGC-5 cells were transfected with GFP-M98K and after 6 hrs cells were either left untreated or treated with BX-795 (0.1μM or 1μM) for 18 hrs. Data represent quantitation of cell death induced by M98K; n = 6. **(F)** M98K-OPTN-induced TFRC degradation in RGC-5 cells is dependent on kinase activity of Tbk1. RGC-5 cells infected with WT-OPTN or M98K-OPTN adenoviruses for 6 hrs were either left untreated or treated with 0.2 μM or 1 μM BX-795 for further 18 hrs. Cell lysates were then subjected to western blotting. Actin was used as a loading control. The numbers below TFRC blot indicate relative TFRC levels after normalization.

So far our results showed that induction of cell death by M98K-OPTN is dependent on phosphorylation at Ser-177 by Tbk1. Tbk1 could, therefore, be an important player in mediating M98K-OPTN-induced TFRC degradation and cell death. To test this possibility, we repressed *Tbk1* by shRNA-mediated knockdown, and observed that M98K-OPTN induced cell death was significantly decreased when cellular Tbk1 levels were reduced (Fig 4A and 4B). Tbk1 knockdown also resulted in reduced caspase-3 activation induced by M98K-OPTN, as shown by western blot for cleaved caspase-3 (Fig 4C). Phosphorylation of M98K-OPTN at Ser-177 was reduced in cells expressing Tbk1 shRNAs (Fig 4D).

Treatment of M98K-OPTN expressing cells with the Tbk1 inhibitor, BX-795, significantly reduced M98K-OPTN-induced RGC-5 cell death (Fig 4E). Since TFRC degradation was a consequence of M98K-OPTN expression, we examined the requirement of Tbk1 activity for M98K-OPTN-induced TFRC degradation. Inhibition of kinase activity of Tbk1 by BX-795 also reduced M98K-OPTN-induced TFRC degradation (Fig 4F). No effect was seen on TFRC levels in WT-OPTN expressing cells (Fig 4F). These results clearly suggest that M98K-OPTN-induced cell death and TFRC degradation depend upon Tbk1-mediated phosphorylation.

### Endogenous Tbk1 Shows Increased Localization to Autophagosomes in M98K-OPTN Expressing Cells

**Fig 5:**
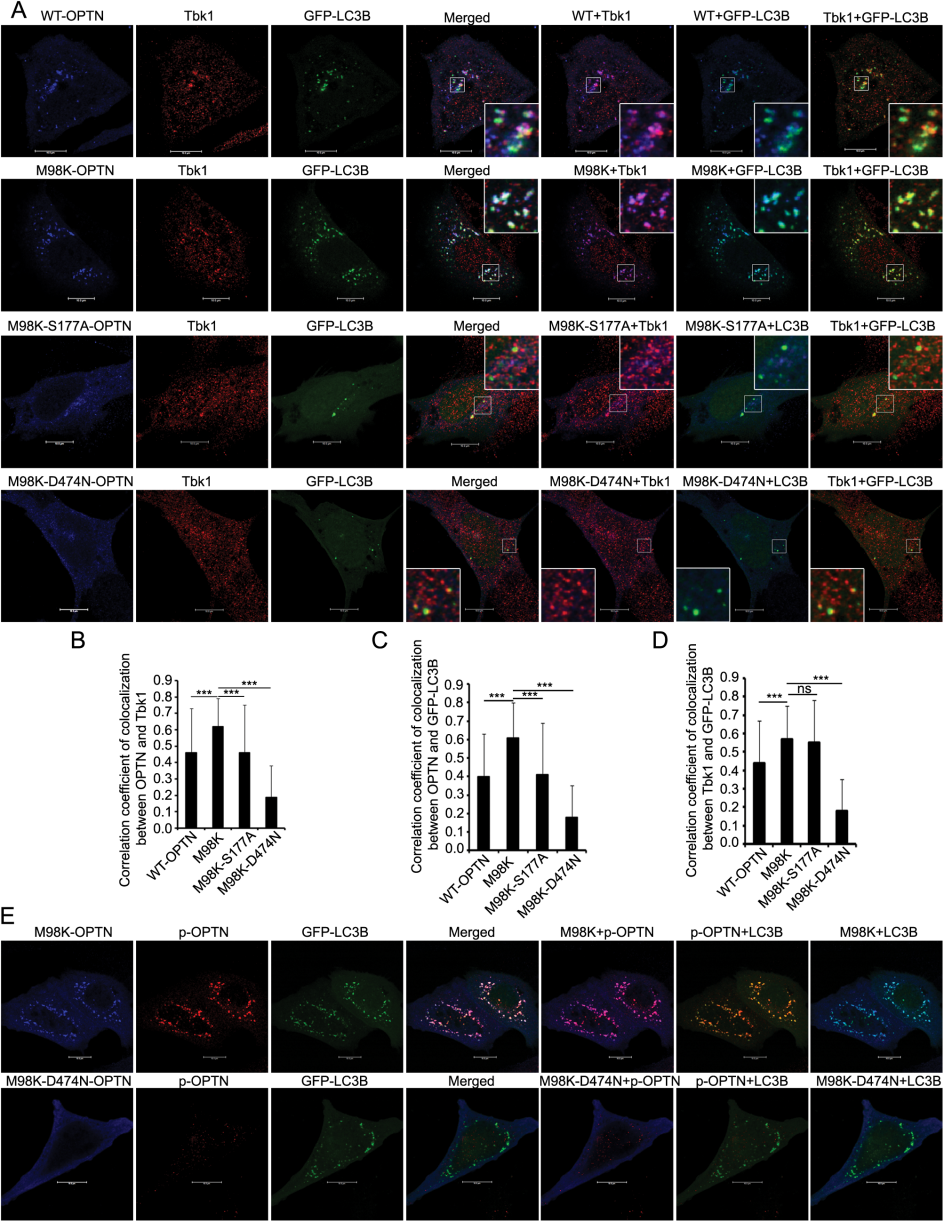
Endogenous Tbk1 shows increased localization to autophagosomes in M98K-OPTN expressing cells. **(A)** Panels show representative confocal images of RGC-5 cells expressing HA-tagged constructs of WT-OPTN, M98K-OPTN, M98K-S177A-OPTN or M98K-D474N-OPTN along with GFP-LC3B, and costained for endogenous Tbk1. Scale bar: 10μm. Magnified areas are shown as insets. **(B)** The graph shows correlation coefficient of colocalization between HA-OPTN and endogenous Tbk1 in GFP-LC3B-positive structures in WT, M98K, M98K-S177A or M98K-D474N expressing cells. **(C)** The graph shows correlation coefficient of colocalization between OPTN and GFP-LC3B in WT, M98K, M98K-S177A or M98K-D474N expressing cells. **(D)** The graph shows correlation coefficient of colocalization between endogenous Tbk1 and GFP-LC3B-positive structures in cells expressing the indicated OPTN mutants. *** < .001. ns, not significant. **(E)** p-S177-M98K-OPTN is preferentially localized to autophagosomes. Representative confocal images show colocalization between p-OPTN and GFP-LC3B in M98K or M98K-D474N expressing RGC-5 cells.

We examined the localization of endogenous Tbk1 in autophagosomes in cells expressing M98K-OPTN. GFP-LC3 and HA-M98K-OPTN were coexpressed in RGC-5 cells, stained for endogenous Tbk1 and examined by confocal microscope. Tbk1 showed colocalization with M98K-OPTN in autophagosomes (GFP-LC3 positive structures) (Fig 5A). Compared to WT-OPTN, M98K-OPTN showed significantly better colocalization with Tbk1 in autophagosomes (Fig 5A and 5B). However, as compared with M98K-OPTN, M98K-S177A and M98K-D474N mutants showed significantly less colocalization with Tbk1 in autophagosomes (Fig 5A–5C). Expression of M98K-OPTN significantly enhanced localization of endogenous Tbk1 in autophagosomes (Fig 5A and 5D). M98K-OPTN, phosphorylated at Ser177, was mostly seen in autophagosomes formed by GFP-LC3 (Fig 5E). These results suggest that phosphorylated form of M98K-OPTN is preferentially recruited to autophagosomes.

### Overexpression of Tbk1 Induces Cell Death in RGC-5 cells, Dependent on Its Kinase Activity

**Fig 6:**
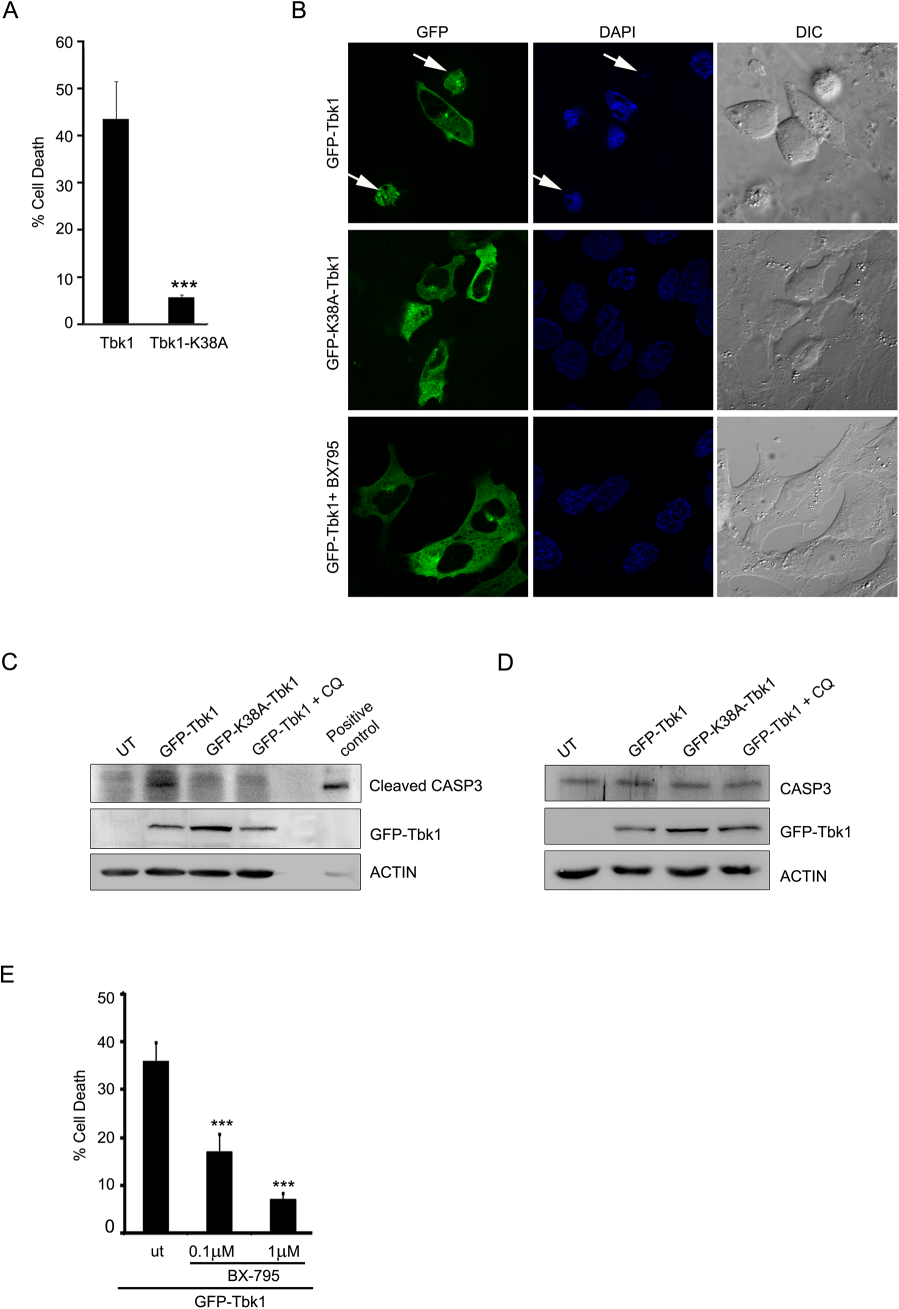
Tbk1 induces cell death in RGC-5 cells dependent on its kinase activity. **(A)** Overexpression of Tbk1 induces cell death while catalytically inactive mutant (K38A) does not induce cell death in RGC-5 cells. Data represent quantitation of cell death induced by GFP-Tbk1. n = 6, ***p < 0.001. **(B)** RGC-5 cells were transfected with GFP-tagged constructs of Tbk1 with or without treatment with BX-795 (1μM) or K38A-Tbk1 mutant. Panels show images captured using 63X objective of Axioplan2 microscope (Zeiss). DAPI and phase panels indicate nuclear and cell morphology respectively. Arrows indicate apoptotic cells. **(C)** Western blot shows cleavage of caspase 3 upon overexpression of GFP-Tbk1 with or without chloroquine (CQ) treatment for 18 hrs or GFP-K38A-Tbk1 mutant. Actin was used as loading control. Cells treated with 10 ng/ml TNF and 20 μg/ml cyclohexamide for 6 hrs were used as a positive control. **(D)** Tbk1 did not change expression of caspase 3. Western blots showing uncleaved caspase 3 upon overexpression of GFP-Tbk1 or K38A-Tbk1 mutant in RGC-5 cells. Actin was used as a loading control. **(E)** Tbk1-induced cell death in RGC-5 cells is dependent on its kinase activity. RGC-5 cells were transfected with GFP-Tbk1 and after 6 hrs, cells were either left untreated (ut) or treated with BX-795 (0.1μM or 1μM) for 18 hrs. Figure shows quantitation of cell death induced by GFP-Tbk1. n = 6, ***p<0.001.

TBK1 plays an important role in several cell signalling pathways, including innate immunity, xenophagic elimination of bacteria, cell growth and proliferation [40]. Cellular TBK1 activity and localization to several signalling complexes depends upon its interacting partners and is tightly regulated. Elevated levels of TBK1 could result in its self activation without an upstream signal. Duplication of the TBK1 gene locus on chromosome 12q14 (GLC1P locus) has been linked to familial NTG in an African-American pedigree and Japanese patients [4, 6]. This prompted us to investigate the effect of increased levels of Tbk1 on cell survival. RGC-5 cells were grown on coverslips and transiently transfected with GFP-Tbk1. After 24 hrs of overexpression in RGC-5 cells, GFP-Tbk1 caused cell death in 41.44 ± 2.47% of expressing cells (Fig 6A and 6B) and also induced activation of the executioner caspase, caspase 3 (Fig 6C). Expression of HA tagged or tag less constructs also induced 35–40% cell death. Tbk1 is a potent inducer of cell death as it was seen that expression of very low levels of plasmid (10 ng) also caused high levels (~40%) of cell death. The kinase-dead mutant of Tbk1, GFP-K38A-Tbk1, was compromised in its ability to induce cell death in RGC-5 (Fig 6A and 6B) or cause cleavage of caspase 3 (Fig 6C), indicating that activation of cell death signalling by Tbk1 required its kinase activity. The level of uncleaved caspase 3 was similar in cells expressing GFP-Tbk1 or its kinase dead mutant (Fig 6D). Inhibition of Tbk1 activity by the inhibitor, BX-795, also prevented Tbk1-induced cell death (Fig 6E). These results suggested that overexpression of Tbk1 induces death of retinal cells which is dependent on its kinase activity.

### Cell Death Induced by Tbk1 in RGC-5 Cells Involves Autophagy

**Fig 7:**
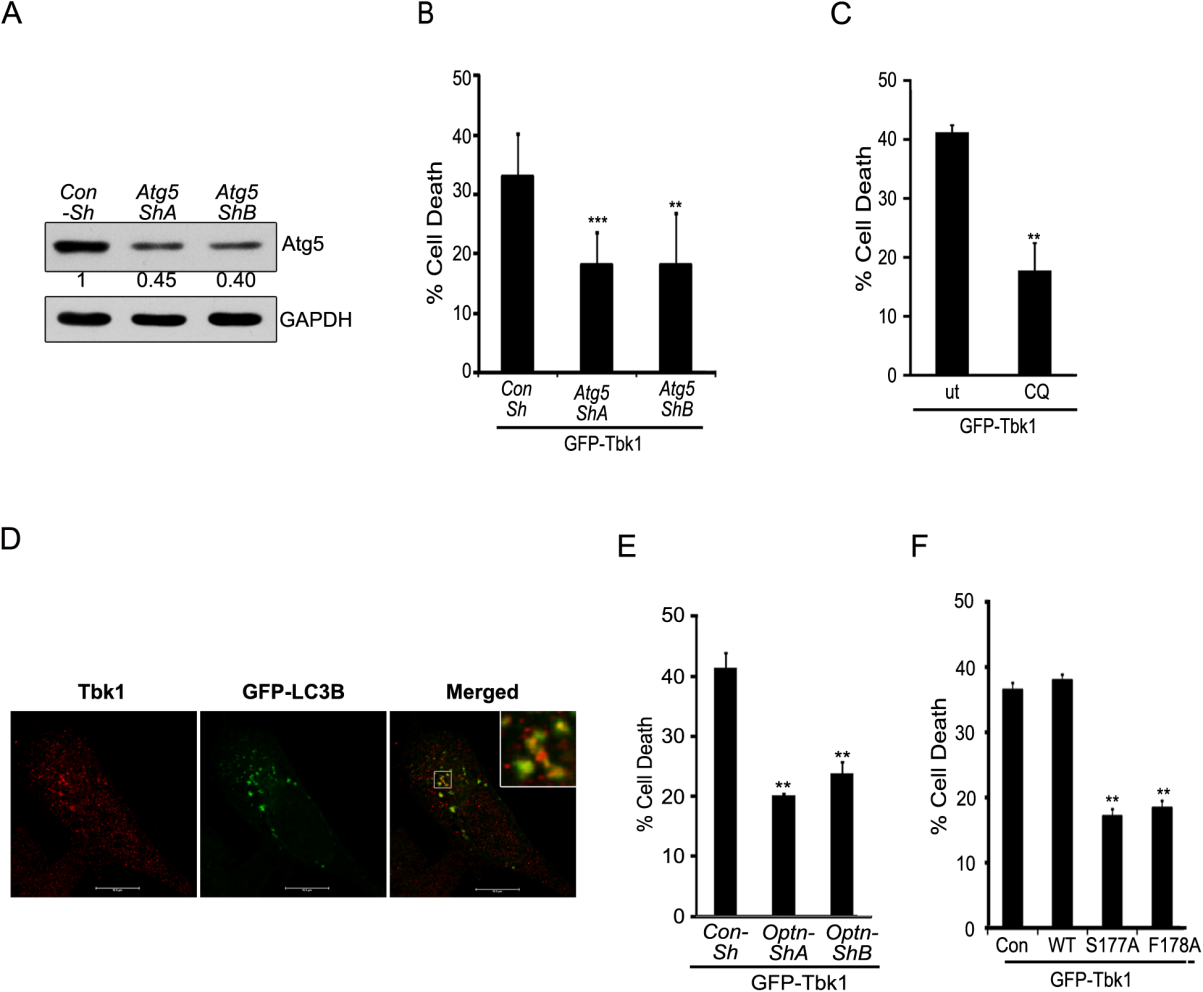
Tbk1-induced cell death in RGC-5 cells is dependent on autophagy and requires OPTN. **(A)** Western blot shows knockdown of Atg5 by shRNAs, shA and shB in RGC-5 cells. GAPDH was used as a loading control. Con-sh, control shRNA. **(B)** Knockdown of Atg5 reduced Tbk1-induced cell death. Data represent quantitation of cell death induced by GFP-Tbk1 with or without Atg5 knockdown. n = 6, ***p < 0.001, **p < 0.01. **(C)** Tbk1-induced cell death depends on lysosomal activity. Data shows quantitation of cell death upon expression of GFP-Tbk1 in the presence or absence (ut) of chloroquine for 18 hrs. n = 6 experiments, **p < 0.01. **(D)** Endogenous Tbk1 shows colocalization with autophagosomes. Representative confocal images of RGC-5 show localization of endogenous Tbk1 with GFP-LC3B-positive structures (autophagosomes). **(E)** Quantitation of cell death induced by GFP-Tbk1 with or without knockdown of OPTN. n = 4, **< 0.01. **(F)** Quantitation of cell death induced by GFP-Tbk1 coexpressed with Con, WT-OPTN, S177A-OPTN and F178A-OPTN. n = 4, **p < 0.01.

TBK1 plays a vital role in autophagy and is also known to phosphorylate autophagy receptors OPTN and p62/SQSTM1 [27, 47]. It was therefore important to test whether Tbk1-induced cell death involves autophagy. Knockdown of *Atg5*, a gene essential for autophagosome formation, by two different shRNAs significantly reduced cell death induced by Tbk1 overexpression (Fig 7A and 7B). The efficacy of these shRNAs to knockdown endogenous Atg5 was confirmed by western blotting (Fig 7A). We have also tested *Atg5* shRNAs to check whether they inhibit autophagy. Using mCherry-GFP-LC3 as reporter, we found that *Atg5* shRNAs significantly inhibit starvation-induced autophagy (S4 Fig). Cell death induced by Tbk1 was significantly reduced when RGC-5 cells expressing GFP-Tbk1 were treated with a lysosomal inhibitor, chloroquine (25μM) (Fig 7C and S5 Fig), which inhibits autophagy flux. Treatment with chloroquine reduced caspase 3 cleavage in Tbk1 expressing cells (Fig 6C). These results suggested the involvement of autophagy in Tbk1-induced cell death. Further support for the involvement of Tbk1 in autophagy was provided by our observation of the colocalization of endogenous Tbk1 with autophagosomes (Fig 7D)

### Tbk1-Induced Cell Death Requires Autophagic Function of OPTN

We observed that M98K-OPTN-induced autophagic cell death requires Tbk1. Since TBK1 and OPTN form a functional complex and interact with each other [27, 37], we examined whether Tbk1-induced cell death required OPTN function. ShRNA mediated knockdown of *Optn* gene significantly reduced Tbk1-induced cell death (Fig 7E). These results suggest that Tbk1-induced cell death was dependent on OPTN. Since Tbk1-induced cell death is mediated by autophagy, we explored the possibility of involvement of autophagic function of OPTN in Tbk1 induced cell death. Coexpression of phospho-defective, S177A, or LC3-binding defective, F178A, mutants of OPTN with GFP-Tbk1 inhibited Tbk1 induced cell death (Fig 7F). Thus, it appears that these two glaucoma-associated proteins, TBK1 and OPTN, regulate each other’s activity to induce autophagy mediated cell death signalling.

## Discussion

M98K variant of OPTN is associated with glaucoma in certain ethnic groups [17–19, 62]. Earlier, we have shown that M98K-OPTN overexpression induces death of retinal cells selectively due to autophagic degradation of TFRC [15]. Here, we explored the molecular mechanism involved in autophagy induction and TFRC degradation by M98K-OPTN in retinal cells. For this purpose, we have used RGC-5 cell line, originally described as a retinal ganglion cell line, which has been recharacterised [33, 58–60]. We think that in spite of not being a retinal ganglion cell line, the RGC-5 cell line, is useful for understanding the molecular mechanisms relevant for glaucoma pathogenesis because it shows properties of neural precursor cells. In addition, expression of E50K-OPTN induces cell death selectively in RGC-5 cells but not in several other cell lines tested [14]. These observations with E50K-OPTN in RGC-5 cells have been confirmed in transgenic mice expressing E50K-OPTN, which show reduced thickness of all cell layers of retina, though development of the eye was normal [32]. Although, it is generally believed that glaucoma is caused by degeneration of retinal ganglion cells, recent studies using ultra high resolution *in vivo* imaging of retina have shown that loss of photoreceptor cone cells also occurs in human glaucoma [63]. Loss of photoreceptor cone cells has been reported in experimental animal models of glaucoma [64, 65]. This topic has been reviewed recently [66].

**Fig 8:**
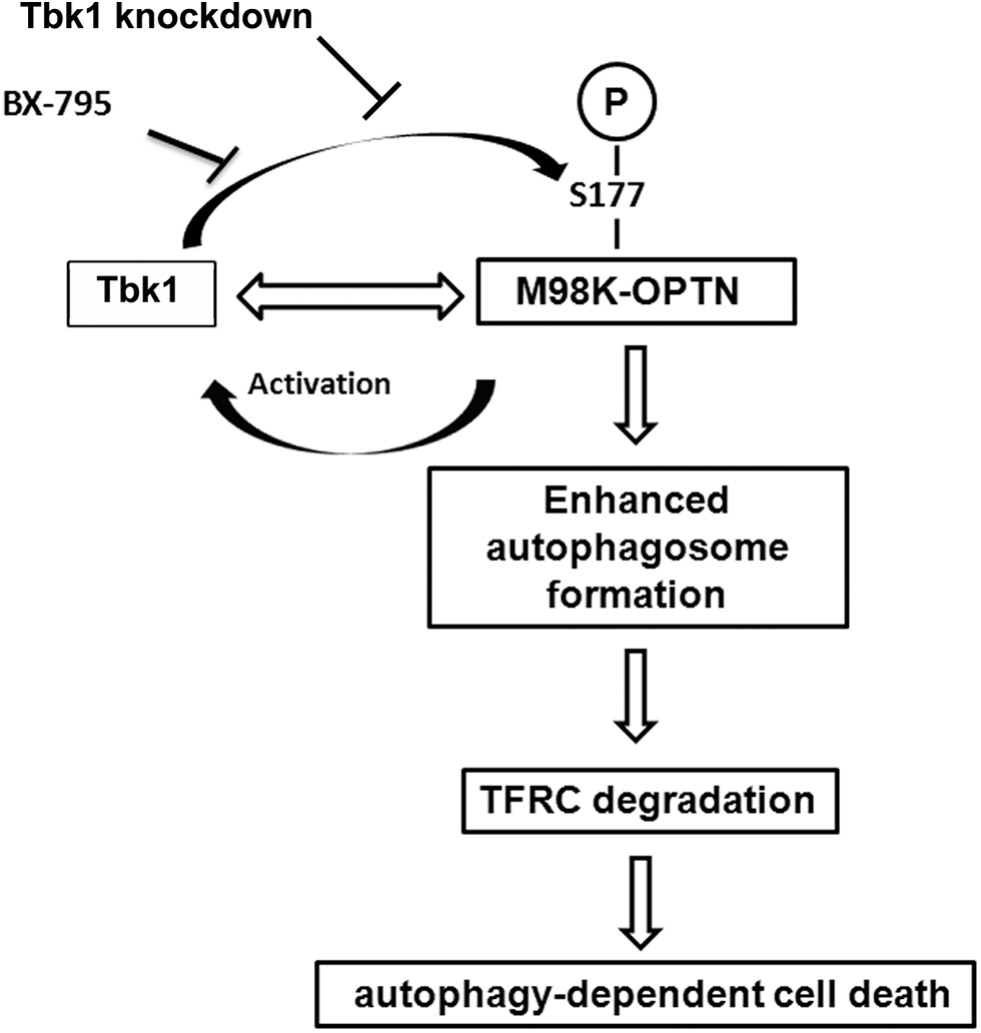
Model showing that how M98K-OPTN functions to induce retinal cell death. M98K-OPTN enhances Tbk1 activation, which in turn leads to enhanced phosphorylation of OPTN at S177, subsequently resulting in increased autophagosome formation and cell death in retinal cells. Inhibition of Tbk1 activity by its chemical inhibitor, BX-795, or by shRNA-mediated knockdown inhibits M98K-OPTN phosphorylation, TFRC degradation and cell death.

Here, we show that phosphorylation of M98K-OPTN at Ser177 by Tbk1 is required for its ability to induce autophagy and cell death in RGC-5 cells (Fig 8). Moreover, M98K-OPTN shows enhanced phosphorylation at Ser177 compared to WT-OPTN that is seen only in retinal cells in which M98K-OPTN induces cell death but not in those cells (HeLa, IMR-32) in which M98K-OPTN does not induce cell death or autophagic degradation of TFRC [15]. These results suggest that enhanced phosphorylation of M98K-OPTN plays a crucial role in retinal cell death induced by this glaucoma–associated variant of OPTN and this might have relevance to glaucoma pathogenesis by M98K-OPTN.

OPTN functions as an autophagy receptor which recruits cargo to the autophagosomes by binding to autophagosomal protein LC3. Our results show that OPTN is required for autophagosome formation and maturation in retinal cells. How does OPTN contribute to autophagosome formation? One possibility is that OPTN contributes to autophagosome formation by functioning as an autophagy receptor. This is supported by our observation that M98K-OPTN, which is phosphorylated more than the WT-OPTN, induces more autophagosome formation, dependent on phosphorylation at S177, a process known to increase interaction of OPTN with LC3 and recruitment to autophagosome. However, it is possible that OPTN contributes to autophagosome formation by some other mechanism also. Recently, it was shown that simultaneous knockdown of three autophagy receptors, OPTN, T6BP and NDP52 leads to reduced autophagosome formation and autophagy, although knockdown of these proteins individually had no significant effect on autophagy in retinal pigment epithelial cells [67]. Therefore, it is likely that in RGC-5 cells, OPTN is particularly important for autophagosome formation.

Enhanced phosphorylation of M98K-OPTN was mediated by Tbk1, a protein kinase that is known to interact directly with OPTN, which is activated upon M98K expression. Ubiquitin binding function of OPTN was required for Tbk1 activation and enhanced phosphorylation of M98K-OPTN. Binding of OPTN to ubiquitinated *Salmonella* leads to TBK1-mediated phosphorylation involved in autophagic clearance of cytosolic *Salmonella* [27]. It has been shown that binding of OPTN with ubiquitinated species increases the activation of Tbk1 and subsequent phosphorylation of OPTN in bone marrow macrophages stimulated with LPS [29]. Similar mechanism could be postulated for UBD-dependent activation of TBK1 by M98K-OPTN.

Recently, duplication of *TBK1* gene locus has been reported in NTG, although no disease-causing mutations in TBK1 have been reported [6]. Duplication of *TBK1* gene locus in NTG patients was associated with increased transcript levels of TBK1 in retina [6, 68]. Gene duplication and increased transcript levels could probably result in increased protein level and activity. Tbk1 overexpression-induced RGC-5 cell death possibly involves autophagy, as shown by the effect of *Atg5* knockdown and lysosomal inhibitor, chloroquine. Using autophagy defective mutants (LC3-binding defective and phosphorylation defective) we showed that the autophagy function of OPTN mediates Tbk1-induced cell death. However, it is possible that Tbk1 overexpression induced retinal cell death involves other autophagy receptors that are known to be phosphorylated by TBK1 in other cell types [27, 47]. How autophagy leads to caspase activation and apoptosis is not clear. One possibility is that enhanced autophagy by M98K-OPTN may lead to degradation of a negative regulator of an initiator caspase resulting in caspase activation. Another possibility is that autophagy may increase expression of a positive regulator of caspase activation. However, these possibilities need to be tested experimentally to establish a molecular mechanism of autophagy mediated caspase activation leading to death of retinal cells.

Earlier studies have described a role for TBK1 in pro-survival and anti-apoptotic signalling [69, 70]. More recently, duplication of TBK1 was shown to result in autophagy in retinal ganglion cells differentiated from induced pluripotent stem cells derived from skin of an NTG patient [71]. In this study, the authors did not examine, but suggested that increased autophagy leads to death of RGCs. In retinal cells, enhanced autophagy is associated with cell death [72, 73]. It is, therefore, possible to conclude that though normal Tbk1 levels function to protect against cell death by inducing autophagy, unscheduled autophagy caused by enhanced Tbk1 levels/ activity leads to retinal cell death. Our results, therefore, indicate that OPTN mutation at M98K or duplication of Tbk1 could result in enhanced Tbk1 activity leading to increase in autophagic flux and cell death dependent on OPTN phosphorylation in retinal cells.

In conclusion, our results show that a glaucoma-associated mutation, M98K, of optineurin enhances its phosphorylation at Ser177 by activating Tbk1 protein kinase. This leads to enhanced recruitment of M98K-OPTN to autophagosomes and increased autophagy flux. Phosphorylation of M98K-OPTN at Ser177 plays a crucial role in autophagosome formation suggesting, therefore, that autophagy receptor function of OPTN, which is dependent on interaction with LC3, is important for autophagosome formation. M98K-OPTN induced retinal cell death is dependent on its phosphorylation at Ser177. We also show that Tbk1, a glaucoma-associated protein, through its catalytic activity, induces death in retinal cells and this requires autophagic function of OPTN. Therefore, this study provides evidence for cross talk between two glaucoma-associated proteins and their dependence on each other to mediate cellular functions; any perturbation in the fine balance of their regulation could lead to cell death.

## Supporting Information

S1 Fig Differentiation and characterization of RGC-5 cells. (TIF)

S2 Fig Phospo-defective mutant of M98K-OPTN, M98K-S177A reduces autophagy flux. (TIF)

S3 Fig Western blots show TBK1 and OPTN levels in RGC-5, HeLa and IMR-32 cells. (TIF)

S4 Fig Knockdown of *Atg5* decreases starvation-induced autophagy. (TIF)

S5 Fig Chloroquine inhibits Tbk1-induced cell death. (TIF)

## Abbreviations

EBBS: Earle’s balanced salt solution
IOP: intraocular pressure
LC3: microtubule-associated protein 1 light chain 3
LIR: LC3-interacting region
NTG: normal tension glaucoma
OPTN: optineurin
POAG: primary open angle glaucoma
RGC: Retinal ganglion cell
shRNA: small-hairpin RNA
TBK1: TANK binding kinase 1
TFRC: transferrin receptor-1
UBD: ubiquitin-binding domain
ULD: ubiquitin-like domain
WT: wild type.
