# Supplementary figures and images for: A Glaucoma-Associated Variant of Optineurin, M98K, Activates Tbk1 to Enhance Autophagosome Formation and Retinal Cell Death Dependent on Ser177 Phosphorylation of Optineurin
Source: PLoS One. 2015 Sep 16;10(9):e0138289. doi: 10.1371/journal.pone.0138289 (PMC4574030; doi:10.1371/journal.pone.0138289)

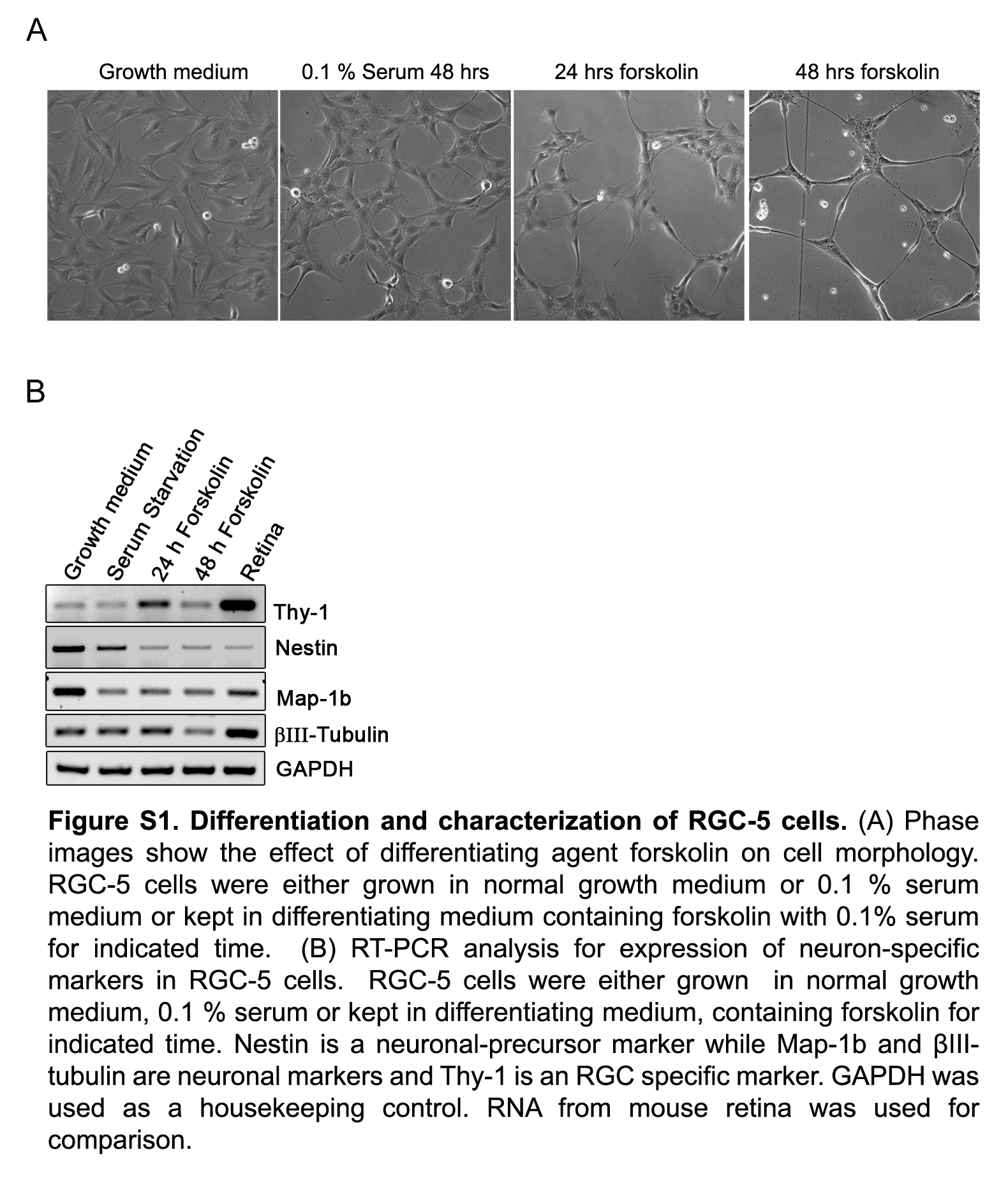

Supplement: S1 Fig — (TIF) [file pone.0138289.s001.tif]

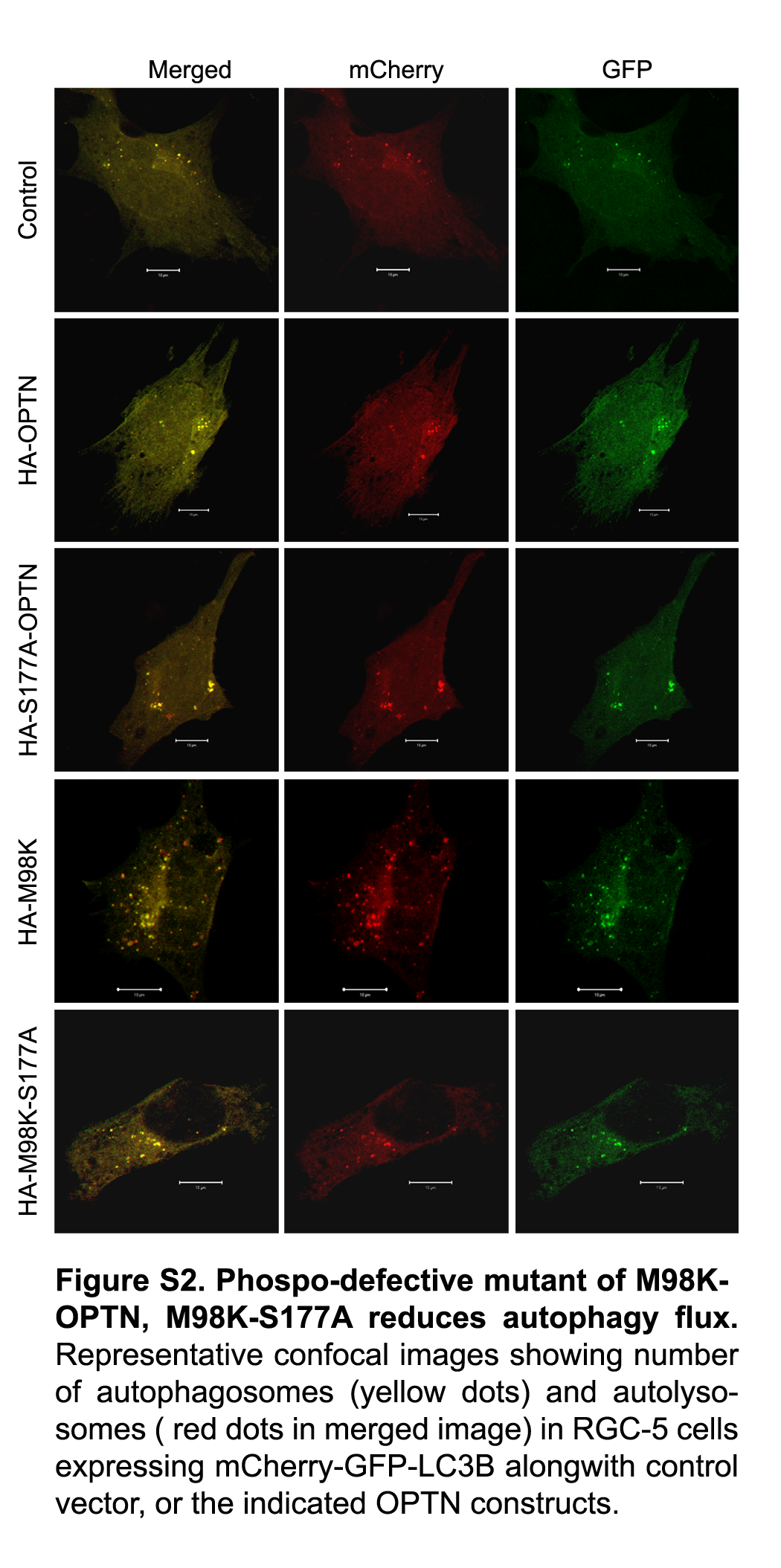

Supplement: S2 Fig — (TIF) [file pone.0138289.s002.tif]

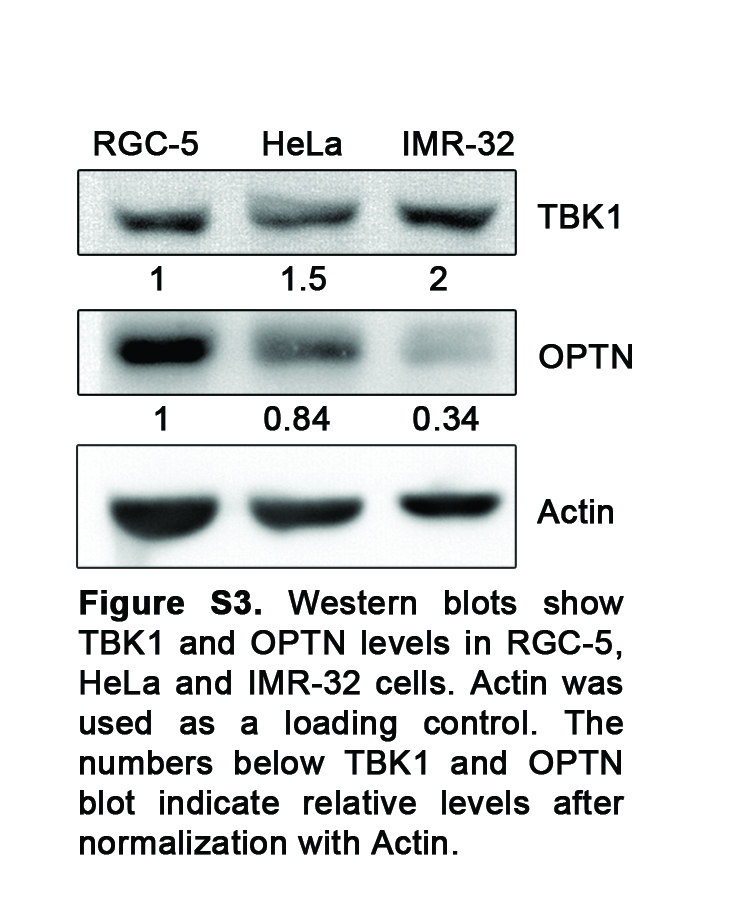

Supplement: S3 Fig — (TIF) [file pone.0138289.s003.tif]

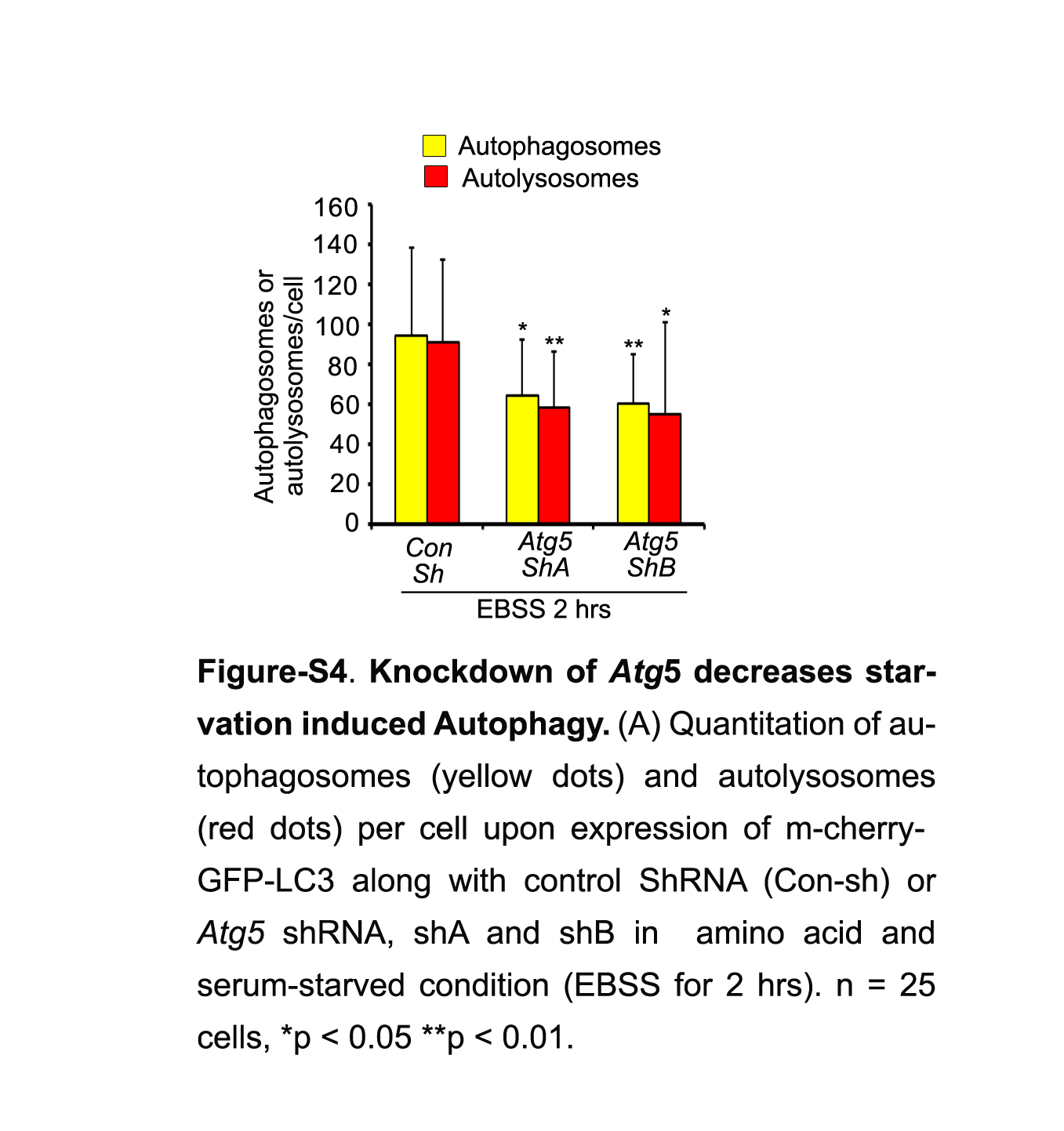

Supplement: S4 Fig — (TIF) [file pone.0138289.s004.tif]

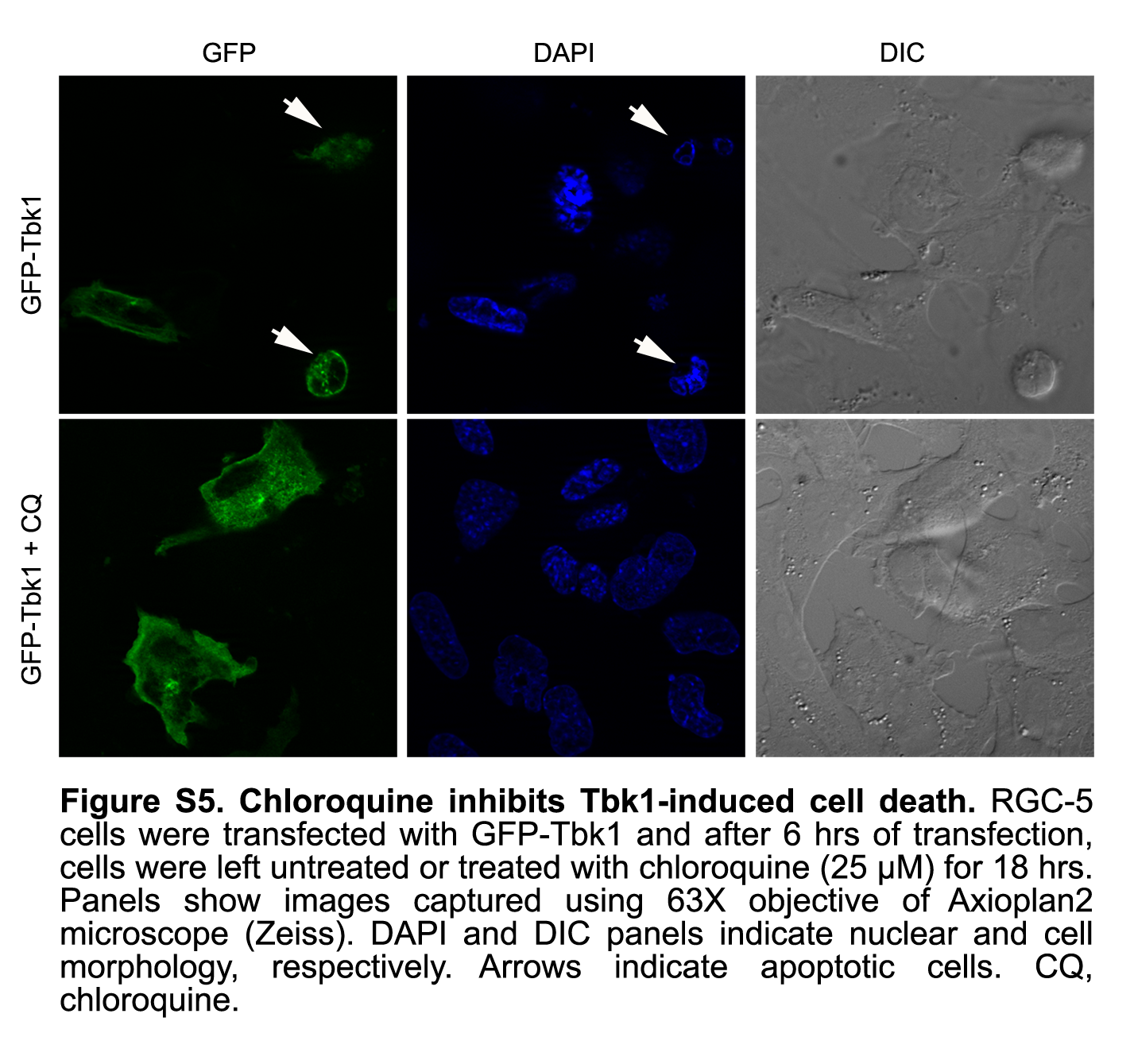

Supplement: S5 Fig — (TIF) [file pone.0138289.s005.tif]
